# The Management of Uterine Arteriovenous Malformations in Obstetrics

**DOI:** 10.7759/cureus.60425

**Published:** 2024-05-16

**Authors:** Linda García-Lima, Bertha Patricia Diaz, Alexandra Bermúdez Rodríguez, Adriana Palacios Macedo Chavolla, Montserrat Malfavon

**Affiliations:** 1 Gynecology, American British Cowdray Medical Center, Mexico City, MEX

**Keywords:** obstetrical hemorrhage, vascular anomalies, postpartum, uterus, arteriovenous malformation

## Abstract

Uterine arteriovenous malformation (AVM) is a rare but serious condition that can cause heavy uterine bleeding. It occurs when abnormal connections form between the arteries and veins in the uterus, leading to significant health complications. Accurate identification and diagnosis are crucial because overlooking or mishandling them can lead to severe, life-threatening bleeding. We present the case of a 30-year-old patient presenting with abnormal uterine bleeding 15 days after she gave birth to her second child. The ultrasound examination showed images suggestive of retained ovuloplacental remnants, so a uterine aspiration was performed, but the patient presented severe vaginal bleeding. Subsequently, magnetic resonance imaging (MRI) was performed, demonstrating the presence of a prominent lesion in the posterior wall of the uterine body with multiple serpentine-like pathways and a signal void suggestive of aberrant vessels corresponding to AVMs. Ergotrate and misoprostol were administered to control the bleeding, and a Bakri balloon was inserted and maintained until the bleeding stopped. We are highlighting this case to emphasize the importance of considering uterine AVM (UAVM) when dealing with abnormal uterine bleeding, even in the postpartum period. Due to its rarity, there is a lack of substantial evidence to guide clinicians in managing this condition.

## Introduction

Abnormal connections between an organ's veins and arteries are known as arteriovenous malformations (AVMs). They can be present in any organ but are more frequently found in the brain, lungs, colon, and soft tissues such as the extremities [1]. Histologically, a uterine AVM (UAVM) is an arteriovenous fistula between the intramural arterial branches of the uterine arteries and the myometrial venous plexus [2,3]. They are a rare medical condition whose incidence is currently unknown and typically occur in females of reproductive age but have been reported as high as 0.63% after delivery or abortion [4]. They are classified as either congenital or acquired [5]. Congenital uterine AVMs result from the abnormal embryologic development of primitive vascular structures, which result in multiple abnormal communications between arteries and veins [2,6,7]. Acquired uterine AVMs can result as a consequence of uterine surgery (cesarean delivery, myomectomy, curettage, etc.) in 96% of the cases, but they can also be formed in cases of endometriosis, endometrial carcinoma, cervical cancer, and gestational trophoblastic disease [5,8,9].

It is crucial to distinguish uterine AVMs from other disorders such as retained ovuloplacental remnants, the subinvolution of the placental bed, and gestational trophoblastic illness. Uterine AVMs are diagnosed when we find a lesion with hypervascular and turbulent flow [5]. The clinical significance of uterine AVMs is mainly related to their potential life-threatening bleeding and the extreme challenge of emergent diagnosis and management once this happens [3]. Early detection and clinical suspicion are critical because in these situations, procedures such as curettage or aspiration may lead to severe hemorrhage and, in some cases, fatal outcomes [10].

## Case presentation

A 30-year-old patient, gravida 2 para 2, with no relevant past medical history, arrived at the hospital with heavy vaginal bleeding. She gave birth to her second child 15 days before, with no further complications reported. The color Doppler ultrasound examination showed a uterus with images suggestive of retained ovuloplacental remnants. In order to establish the diagnosis, uterine aspiration was performed, but the patient presented extensive vaginal bleeding (1000 mL). Once proper consent was obtained, she was moved to the operating room. There, an ultrasound-guided uterine aspiration procedure was carried out. Intramuscular 0.2 mg of Ergotrate and 600 mcg of rectal misoprostol were administered, a hemogram was taken (Table 1), and the uterine tone was confirmed; however, on ultrasound scan, the images suggestive of remnants persisted, so a Bakri balloon was inserted with 50 cc.

**Table 1:** Hemogram test taken during the surgical procedure. Hgb, hemoglobin; Hct, hematocrit; MCV, mean corpuscular volume; MCH, mean corpuscular hemoglobin; PLT, platelet; NEUT, neutrophil; LYMPH, lymphocyte

Complete blood count			
Parameter	Value	Unit	Reference value
WBC	10.81	10^3^/µL	4.80-11.0
RBC	2.31	10^6^/µL	4.0-5.60
Hgb	6.9	g/dL	13.5-16.5
Hct	21.3	%	38.5-47.0
MCV	92.2	fL	80.0-99.0
MCH	29.9	Pg	27.0-33.0
PLT	160	10^3^/µL	150-450
NEUT	56	%	
LYMPH	37.30	%	

**Figure 1:**
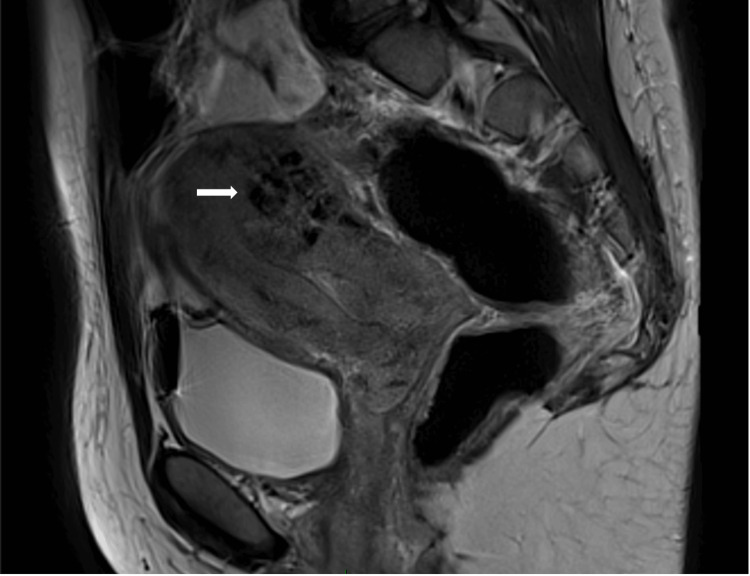
Sagittal T2-weighted sequence image showing multiple serpentine-like pathways with signal void suggestive of aberrant vessels in the posterior wall of the uterine body, with areas of interposed myometrium (white arrow) visualized.

Once the bleeding was controlled, a magnetic resonance imaging (MRI) was requested. The MRI reported a prominent lesion in the posterior wall of the uterine body with multiple serpentine-like pathways with signal void suggestive of aberrant vessels (Figure 1).

**Figure 2:**
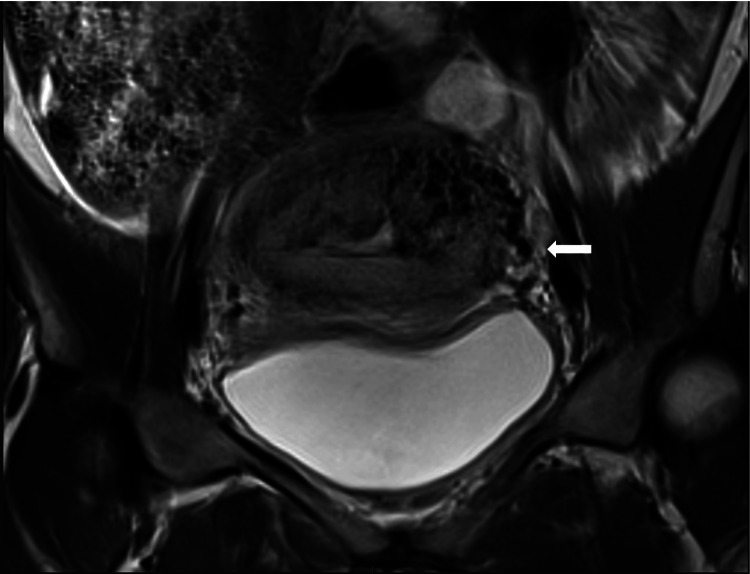
Coronal image showing the ectasia of the branches of the left uterine artery, with communication to the nest of aberrant vessels in the uterine body (white arrow).

**Figure 3:**
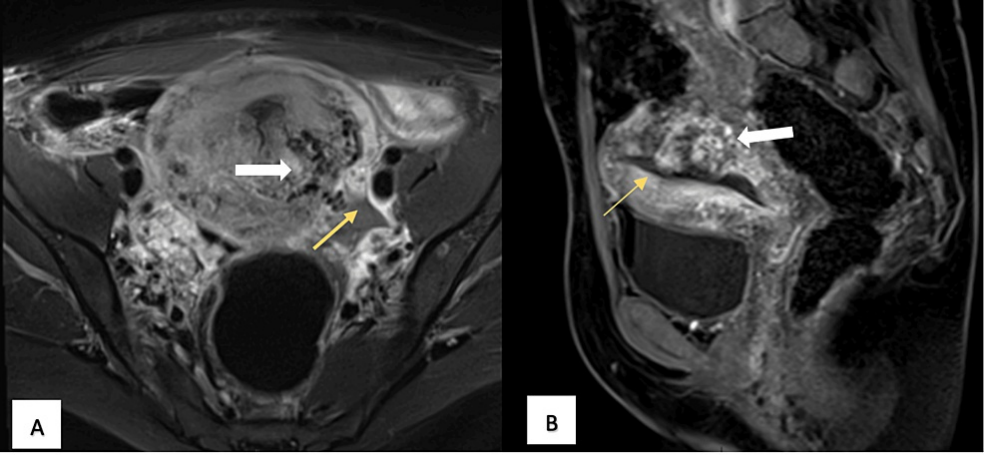
(A) Axial image, T1-weighted sequence FAT SAT with gadolinium in the early phases of dynamic acquisition, showing an enhancement of the heterogeneous appearance of the structures of the vascular nidus (white arrow); the ectasia of the pelvic vascular structures is confirmed (yellow arrow). (B) Sagittal image, T1-weighted sequence FAT SAT with gadolinium in the late phases of dynamic acquisition, showing the enhancement of aberrant vascular structures (white arrow) with the projection of vascular structures toward the endometrial cavity (yellow arrow). FAT SAT: fat saturation

That lesion shows vascular pathways through the endometrial surface with an irregular contour (Figures 2, 3).

The total blood loss during the surgical procedure was 1000 mL more, and two units of packed red cells were infused during the surgery. No other medical or surgical treatment was required. The patient entered the intensive care unit and was discharged one week later in good condition. The patient was re-evaluated two weeks later, without transvaginal bleeding events and with adequate evolution.

## Discussion

AVMs are rare in the uterus and are generally refractory to usual treatment, presenting as severe menstrual bleeding. To date, less than 100 cases with this condition have been reported, and the first one was initially described in 1926 by Dubreil and Loubat as "cirsoid aneurysm of the uterus" [5,11].

Congenital uterine AVMs are thought to develop when the embryonic capillary network does not differentiate properly, leading to abnormal connections between arteries and veins. They tend to have multiple feeding arteries and numerous large draining veins [7,12]. Recent studies revealed that mutations in the *RASA-1* gene on 5q13-22 are responsible for many congenital capillary malformations and AVMs [13,14]. Nevertheless, most uterine AVMs are sporadic. On the other hand, acquired uterine AVMs arise as a complication of uterine interventions. The main associated procedures are curettage, cesarean sections, and myomectomies. Also, they can overlap in the presentation and appearance of other pathologies related to pregnancy and uterine trauma, such as the subinvolution of the placental bed, retained products of conception, incomplete miscarriage, and gestational trophoblastic disease [10,15]. A precise diagnosis is vital because the typical treatment for these complications (usually dilation and curettage) is not recommended in AVMs due to the potential risk of causing massive hemorrhage and death [10].

It is believed that iatrogenically, abnormal physiological repair mechanisms are carried out in these tissues. If the repair process begins when the vessels are juxtaposed and the capillary plexuses are not involved, a fistula can be created when physiological circulation resumes [1]. In the case of congenital AVMs, they originate from abnormal differentiation in the fetal capillary plexus, which is why they can clinically occur during early menarche and may exhibit numerous vascular connections while also potentially infiltrating other organs [11,16].

The clinical presentation of uterine AVMs is variable; the classical clinical feature is intermittent, heavy vaginal bleeding, which can be life-threatening but can also be asymptomatic [17]. In the case of pregnant patients, uterine AVMs must be suspected if obstetric hemorrhage presents and there is no adequate response despite standard treatment approaches [16]. Some patients may also present with recurrent pregnancy loss, incidental pelvic masses in addition to menorrhagia, anemia, and chronic pelvic pain [4,11]. It is believed that up to 50% of cases with acquired AVMs will require blood transfusions [1]. In severe cases, congestive heart failure and a wide shunt may be encountered, although this is not typical [11]. Other symptoms exhibited by patients with uterine AVMs include polyuria and dyspareunia with pelvic pain [1].

Historically, diagnosis was made through histopathology with the hysterectomy specimen; however, nowadays, we have more tools available for a more precise analysis [1]. Initial diagnosis is performed using endovaginal Doppler ultrasound, as it is considered the basic study in gynecological patients [11]. On ultrasound, it appears as thickening and multiple irregular, anechoic spaces in the myometrium, and upon Doppler application, it exhibits a characteristic multidirectional turbulent flow with high velocity and low resistance, displaying a mosaic color pattern [10]. When there is uncertainty, MRI or contrast-enhanced angiotomography, which is the gold standard, is conducted, serving both diagnostic and therapeutic purposes and enabling embolization to be performed simultaneously [10,16]. Through magnetic resonance (MR) angiography, we can observe a serpiginous flow and disruption in the myometrial-endometrial interface zone. On angio-CT, it can be visualized as a hypervascularized mass in the myometrium with intense enhancement in the arterial phase and increased uterine flow [18]. Currently, digital subtraction angiography is considered the best diagnostic option, allowing for a more defined characterization of lesion size, filling and emptying intervals, as well as the relationship with pelvic vasculature, facilitating timely embolization [16].

The management of uterine AVMs largely depends on the patient's context and should be individualized based on hemodynamic status, amount of bleeding, and fertility desires [16]. A recent meta-analysis involving 121 females receiving medical treatment for uterine AVM found that the overall success rate of medical management, utilizing progestins, gonadotropin-releasing hormone agonists (GnRHa), methotrexate, combined hormonal contraception, uterotonics, danazol, or a combination thereof, was 88% [4]. However, none of those medical treatments were performed on patients with unstable hemodynamics, so their use may be reasonable only in very well-selected patients. In our case, we also introduced a Bakri balloon to put pressure on the uterine walls controlling the bleeding. Hysterectomy is considered the gold standard in this pathology. However, since fertility-sparing treatment options are of particular interest, uterine artery embolization (UAE) is often considered a first-line uterine-sparing treatment for symptomatic uterine AVMs achieving success rates of up to 91% after two procedures [1,19]. Treating UAVM with embolization has fewer complications than surgical management. In addition, new embolization agents and hyperselective technical procedures can preserve reproductive capacity as demonstrated by the several successful pregnancies and deliveries after bilateral UAE for UAVM reported in the literature [18]. This treatment is possible because of the collateral circulation present in the uterus, which allows for subsequent pregnancy if desired by the patient. In this case, although we have the availability of this kind of treatment in our center, this treatment was not further necessary because the bleeding was controlled. Some disadvantages of invasive treatment such as UAE include its limited availability in all centers, its costliness, and its occasional association with premature ovarian failure and uterine synechiae [4,20].

One of the reasons for writing this article is the current lack of consensus regarding the management of this uterine pathology and the discrepancy between active and expectant management. Medical management offers several advantages over procedures such as UAE. It is more accessible, less invasive, and cheaper and potentially carries fewer long-term effects on fertility. However, the reported results have been inconsistent, and there is a lack of standardized approaches. Furthermore, there is insufficient research analyzing cases of subsequent pregnancies following a conservative and expectant treatment versus an invasive one such as embolization [18].

## Conclusions

The diagnosis and treatment of this rare condition are still considered challenging due to the lack of evidence and the absence of established clinical practice guidelines for decision-making. Patient history can help us distinguish congenital from acquired AVMs because the imaging features of both may be quite similar. Ultrasound with color Doppler is the preferred imaging method for suspected uterine AVM, and it can be confirmed noninvasively with an MRI. However, angiography is still the preferred imaging method when there is a strong suspicion of AVM in a patient who might undergo embolization as treatment.

Once the correct diagnosis of a uterine AVM is made, quickly identifying that a traumatic uterine AVM is the source of bleeding is crucial for patient care. In such cases, using uterine instrumentation such as dilatation and curettage (D&C) could worsen the condition. Medical management is a reasonable approach in well-selected patients who are hemodynamically stable. Despite reports of future pregnancies occurring after the medical management of uterine AVM, there is limited information available for counseling regarding the treatment's effects on subsequent fertility and pregnancy outcomes. For patients experiencing hemodynamic instability or severe uterine bleeding, UAE or other procedural interventions will still be the preferred treatment, but it is important to consider availability, costs associated with treatment, prolonged hospitalization, and future risk of reproductive complications. Since there are no established clinical guidelines to direct clinicians in choosing patients for treatment, decisions should be personalized and made through discussions between patients and their healthcare providers.
